# Double Burden of Malnutrition Among Pregnant Women in Rural Jharkhand: Evidence From a Cross-Sectional Study

**DOI:** 10.7759/cureus.74692

**Published:** 2024-11-28

**Authors:** Shailesh S Hembrom, Manisha Kujur, Vidya Sagar, Prerna Anand, Surendra Sahu, Mary P Murmu, Kumari Asha Kiran

**Affiliations:** 1 Community Medicine, Rajendra Institute of Medical Sciences, Ranchi, IND; 2 Community Medicine, Medini Rai Medical College, Palamu, IND; 3 Community Medicine, Sheikh Bhikhari Medical College and Hospital, Hazaribag, IND; 4 Pathology, Rajendra Institute of Medical Sciences, Ranchi, IND

**Keywords:** bmi, diet pattern, maternal malnutrition, minimum dietary diversity for women (mdd-w) score, nutritional status

## Abstract

Introduction: The nutritional status of pregnant women is a very important aspect of maternal and antenatal care, as malnutrition is detrimental to both the mother and the foetus. This study tries to assess the scale of the double burden of malnutrition in a rural setting in India.

Methods: A cross-sectional study was conducted on 337 pregnant women to assess the nutritional status of pregnant women using Body Mass Index and dietary intake.

Results: Overall, 21.4% of women were underweight, 14.8% were overweight, 0.9% were obese, and 62.9% of women were in the normal weight range based on Body Mass Index. Family type, dietary habits, and community practices were found to be significant determinants of nutritional status among pregnant women.

Conclusion: Nearly a third of the pregnant women were found to be malnourished, indicating the significant impact of the double burden of malnutrition. Lifestyle changes such as dietary improvement need to be emphasised to enhance maternal nutritional status during antenatal care.

## Introduction

Malnutrition refers to deficiencies, excesses, or imbalances in a person’s intake of energy and/or nutrients. In all its forms, it includes undernutrition (wasting, stunting, underweight), inadequate vitamins or minerals, overweight, obesity, and resulting diet-related noncommunicable diseases. Although malnutrition affects people of all age groups and sexes, women, infants, children, and adolescents are at particular risk for the same [1]. Subsequently, pregnant women can be considered a special high-risk group as more than one life is at stake at the same time.

Resulting after the successful fertilisation of a sperm and an ovum and the subsequent implantation of the embryo, pregnancy is an incredible mechanism provided by nature to ensure the safety, well-being, and development of future off springs of a species. The various physiological changes that take place in the female body during pregnancy not only protect the fetus but also provide the necessary conditions for its development until parturition.

One of these necessary conditions is providing nutrition to the foetus. The foetus derives its nutritional requirements from the pregnant woman through the placental blood supply. Consequently, the proper development of the foetus is directly dependent on the nutritional status of the pregnant woman [2].

The nutritional status of an adult is determined primarily by calculating the Body Mass Index (BMI), also known as the Quetelet’s Index. The WHO has classified the nutritional status of an individual based on the BMI as follows: Underweight, Normal Weight, Overweight, and Obese. In adults, BMI has emerged as a risk indicator for some common conditions related to overweight and obesity, including cardiovascular diseases, high blood pressure, osteoarthritis, some cancers, and diabetes [3].

The simultaneous presence of both undernutrition and overnutrition in a population is known as the Double Burden of Malnutrition (DBM) [4]. Overweight/obesity among pregnant women is associated with complications such as gestational diabetes, pre-eclampsia, gestational hypertension, postpartum haemorrhage, instrumental delivery, cesarean delivery, low birth weight, preterm birth, congenital malformation, large-for-gestational-age babies and perinatal death [5]. On the other hand, underweight pregnant women are more likely to have complications, such as low birth weight, small for gestational age, preterm birth, and neonatal mortality [5]. According to a recent report by The United Nations International Children's Emergency Fund (UNICEF), more than one billion adolescent girls and women suffer from undernutrition (including underweight and short height), deficiencies in essential micronutrients, and anaemia, with devastating consequences for their lives and well-being [6]. Additionally, the global prevalence of obesity among women increased from 6·4% to 14·9% over the past four decades [7].

In the Indian context, according to the National Family Health Survey (NFHS)-5 (2019-21) [8], 19% of women aged 15-49 years have a BMI of less than 18.5 (classified as thin), 18% have a BMI more between 25 and 29.9 (classified as overweight) and 6.4% have BMI more than 30.00 (classified as obese). Adding up the data, we have 43% of women who do not fall in the normal weight category. In the state of Jharkhand, 26.2% of women fall under the thin category, while 11.9% fall under the overweight or obese category. The same data for pregnant women is missing, as mentioned earlier.

The nutritional status of pregnant women based on BMI has been a largely neglected domain in India. The NFHS provides data on the nutritional status of adults based on BMI, but the same for pregnant women is missing. When it comes to pregnancy, most of the attention is being rightfully directed toward the status of anaemia, and the resultant interventions are focused on improving the level of Haemoglobin in pregnant women. But, at the same time, nutritional status during pregnancy based on BMI should not be neglected as currently we are facing the dual burden of undernutrition as well as over-nutrition. Maternal undernutrition contributes to poor foetal growth, low birthweight (LBW), and short- and long-term infant morbidity and mortality [9]. On the other hand, maternal obesity is a risk factor for miscarriages, foetal malformations, intra-uterine foetal deaths (IUFD), and stillbirths [2].

Adult malnutrition is an important public health problem that contributes to significant morbidity and mortality in the community. In recent times, malnutrition has emerged as a double-edged sword, with both undernutrition and over-nutrition on the rise. This emerging public health problem has been termed as the double burden of malnutrition (DBM) [10]. Malnutrition during pregnancy has been a highly neglected aspect of adult nutrition. Not only has it been ignored as an essential part of NFHS (National Family Health Survey), but health programs related to Reproductive and Child Health (RCH) services also do not include it as part of the Antenatal Care (ANC) register.

This study sought to investigate the extent of malnutrition among pregnant women residing in a rural block of Ranchi district, Jharkhand. By assessing the nutritional status within this population, the research aims to highlight critical health challenges faced by expecting mothers in under-resourced areas, contributing to the broader understanding of maternal health disparities in rural India. This could further help in making policies that tackle the issue of maternal malnutrition and help in reducing the adverse birth outcomes that come with it.

## Materials and methods

This study was a community-based cross-sectional study conducted at seven health subcenters (Ayushman Arogya Mandir) coming under the rural field practice area of a medical college in Ranchi district, Jharkhand, India. Sample size was calculated using the formula $n = \frac{Z^{2}\times p\times q}{d^{2}}$, where n = sample size, Z = standard normal deviate (For 95% CI, the value was taken as 1.96), p = prevalence = 32% [11], q = (100-p) = 68%, d = allowable error (also called as precision) = 5%. The final sample size came out to be 334. For the purpose of the study, a total of 337 pregnant women were enrolled.

From each of these seven subcenters, 48 pregnant women were selected at random from the ANC register and enrolled for the study after obtaining informed consent. Pregnant women who were willing to participate in the study were included in the study while those pregnant women who were suffering from any chronic disease, namely hypertension, diabetes mellitus, and hypothyroidism were excluded from the study.

A pre-tested semi-structured questionnaire was used to collect data from all the pregnant women at the respective health subcenter after obtaining informed consent. A questionnaire comprising questions pertaining to the variables was used for data collection. Data collection from the subjects was done in a comfortable environment after written informed consent was taken from them. Questionnaires were filled out by the researcher based on the responses of questions asked to the subject.

The data collected included sociodemographic variables, dietary habits, and height and weight of the study participants. The height was measured using a Seca 213 portable stadiometer (Seca, Hamburg, Germany), while weight was measured using a Beurer PS 160 Digital Bathroom Scale (Beurer India Pvt. Ltd., Gurugram, India). Body mass index was calculated and classified according to the guidelines provided by WHO [3]. The dietary profile was focused on the dietary habits of the individuals, in particular their choice of diet, and dietary diversity score based on the Minimum Dietary Diversity for Women (MDD-W) [12].

Data was entered in a Microsoft Excel for Mac, Version 16.91 (Microsoft Corporation, Redmond, USA) spreadsheet after the generation of the proper template. Height and weight were used to calculate BMI for each participant and further classification was done based on WHO guidelines [3]. Data analysis was done using jamovi software version 2.3 (The jamovi project, Sydney, Australia). Frequency tables were generated to see the distribution of the variables. Pearson's chi-square test was applied to see the association between categorical variables. Logistic regression analysis was done to calculate the odds ratio of outcomes.

## Results

Profile of the study participants

A total of 337 pregnant women participated in the study. The mean age of participants was 24.1±3.61 years. The majority of the participants (70.6%) belonged to the 18-25 years age group. Most of the participants were followers of Hindu religion (41.8%). With regards to ethnicity, 38.9% of the participants were tribal women, while the remaining were from a non-tribal background. Most of the women were educated up to matriculate level (31.5%). The majority of the women were housewives (88.7%). Most of the participants belonged to lower-middle-class families (class IV), according to the updated BG (Brahm Govind) Prasad scale for the year 2024 (40.9%) [13] (Table 1). Around four-fifths (79.5%) of the women were non-vegetarians. The majority of women (58.5%) had an adequate dietary diversity score based on the Minimum dietary diversity-Women (MDD-W) scale based on a 24-hour recall of their diets (Table 1).

**Table 1:** Profile of the study participants BG: Brahm Govind, MDD-W: Minimum dietary diversity-Women: Based on 10 food groups, adequate when an individual consumes food from at least 5 food groups, inadequate when she consumes food from less than 5 groups over the past 24 hours, data collected through 24-hour recall method

Socio-demographic variables		Frequency (n = 337)	Percentage
Age group	18-25 years	238	70.6
	26-30 years	86	25.5
	31-35 years	12	3.6
	36-40 years	1	0.3
Religion	Hindu	141	41.8
	Sarna	101	30
	Muslim	93	27.6
	Christian	2	0.6
Ethnicity	Non-tribal	206	61.1
	Tribal	131	38.9
Educational status	Post-graduate	8	2.4
	Graduate	30	8.9
	Intermediate	94	27.9
	Matriculate	106	31.5
	Up to high school	85	25.2
	Literate without formal education	8	2.4
	Illiterate	6	1.8
Occupation	Housewife	299	88.7
	Student	10	3
	Employed with private firm	17	5
	Teacher	6	1.8
	Farmer	1	0.3
	Labor	2	0.6
	PRI member	1	0.3
	Anganwadi worker	1	0.3
Family type	Joint	286	84.9
	Nuclear	51	15.1
Socio-economic status based on updated BG Prasad Classification Scale (2024)	Class I (Upper)	8	2.4
	Class II (Upper middle)	37	11
	Class III (Middle)	121	35.9
	Class IV (Lower middle)	138	40.9
	Class V (Lower)	33	9.8
Dietary variables			
Dietary pattern	Vegetarian	69	20.5
	Non-vegetarian	268	79.5
Dietary diversity score based on MDD-W	Adequate	197	58.5
	Inadequate	140	41.5

Nutritional status of study participants based on BMI

**Figure 1:**
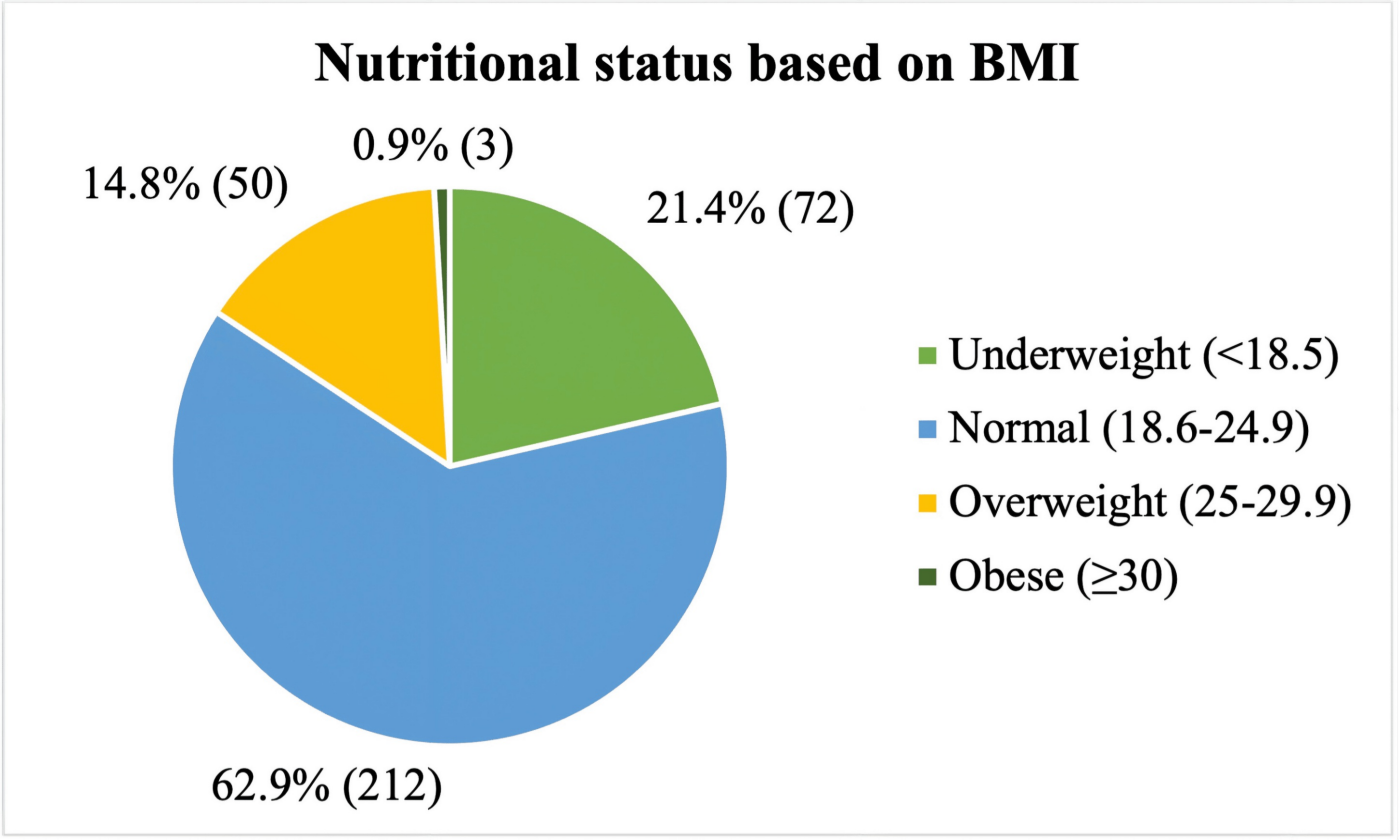
Pie chart showing distribution of study participants according to their nutritional status based on BMI (n = 337) BMI: Body mass index

The mean values of height, weight, and BMI of the participants were 1.51±0.05 meters, 48.7±8.3 kg, and 21.5±3.4 kg/m^2^ respectively. On the basis of Body Mass Index (BMI), 21.4% (72) of women were found to be underweight, while 14.8% (50) and 0.9% (3) women were found to be overweight and obese respectively. Nearly one-third (62.9%) of the women had a BMI within the normal range (Figure 1).

Association of participants’ profile with malnutrition

The majority of women who were malnourished were housewives (88.8%) belonging to the 18-25 years age group (68.0%) coming from a non-tribal background (61.1%). Most of them were followers of the Hindu religion (40.8%), belonging to lower middle-class families (40.8%). Just under a third (29.6%) of women were educated up to the matriculate level. Up to nine out of 10 (90.4%) malnourished women were residing in a joint family setup, which was the only statistically significant finding among all the socio-demographic variables (Table 2).

**Table 2:** Association of study variables with maternal malnutrition (n=337) Test of significance: Pearson's chi-square test BMI: Body mass index, BG: Brahm Govind, MDD-W: Maternal dietary diversity-Women * Statistically significant

Socio-demographic variables		Nutritional status based on BMI			
		Normal (n=212)	Malnutrition (n=125)	Total (n=337)	p-value
Age group	18-25 years	153 (72.2%)	85 (68.0%)	238 (70.6%)	0.713
	26-30 years	51 (24.1%)	35 (28.0%)	86 (25.5%)	
	31-35 years	7 (3.3%)	5 (4.0%)	12 (3.6%)	
	36-40 years	1 (0.5%)	0	1 (0.3%)	
Religion	Hindu	90 (42.5%)	51 (40.8%)	101 (30.0%)	0.489
	Sarna	66 (31.1%)	35 (28.0%)	141 (41.8%)	
	Muslim	54 (25.5%)	39 (31.2%)	93 (27.6%)	
	Christian	2 (0.9%)	0	2 (0.6%)	
Ethnicity	Non-tribal	127 (59.9%)	79 (63.2%)	206 (61.1%)	0.549
	Tribal	85 (40.1%)	46 (36.8%)	131 (38.9%)	
Educational status	Post-graduate	5 (2.4%)	3 (2.4%)	8 (2.4%)	0.919
	Graduate	17 (8.0%)	13 (10.4%)	30 (8.9%)	
	Intermediate	62 (29.2%)	32 (25.6%)	94 (27.9%)	
	Matriculate	69 (32.5%)	37 (29.6%)	106 (31.5%)	
	Up to high school	51 (24.1%)	34 (27.2%)	85 (25.2%)	
	Literate but no formal education	4 (1.9%)	4 (3.2%)	8 (2.4%)	
	Illiterate	4 (1.9%)	2 (1.6%)	6 (1.8%)	
Occupation	Housewife	188 (88.7%)	111 (88.8%)	299 (88.7%)	0.501
	Student	5 (2.4%)	5 (4.0%)	10 (3.0%)	
	Employed with private firm	12 (5.7%)	5 (4.0%)	17 (5.0%)	
	Teacher	4 (1.9%)	2 (1.6%)	6 (1.8%)	
	Farmer	1 (0.5%)	0	1 (0.3%)	
	Labor	2 (0.9%)	0	2 (0.6%)	
	PRI member	0	1 (0.8%)	1 (0.3%)	
	Anganwadi worker	0	1 (0.8%)	1 (0.3%)	
Family type	Joint	173 (81.6%)	113 (90.4%)	286 (84.9%)	0.030*
	Nuclear	39 (18.4%)	12 (9.6%)	51 (15.1%)	
Socio-economic status based on updated BG Prasad Classification Scale (2024)	Class I (Upper)	4 (1.9%)	4 (3.2%)	8 (2.4%)	0.890
	Class II (Upper middle)	22 (10.4%)	15 (12.0%)	37 (11.0%)	
	Class III (Middle)	79 (37.3%)	42 (33.6%)	121 (35.9%)	
	Class IV (Lower middle)	87 (41.0%)	51 (40.8%)	138 (40.9%)	
	Class V (Lower)	20 (9.4%)	13 (10.4%)	33 (9.8%)	
Dietary variables					
Dietary pattern	Vegetarian	43 (20.3%)	26 (20.8%)	69 (20.5%)	0.910
	Non-vegetarian	169 (79.7%)	99 (79.2%)	268 (79.5%)	
Dietary diversity score based on MDD-W	Adequate	134 (63.2%)	63 (50.4%)	197 (58.5%)	0.021*
	Inadequate	78 (36.8%)	62 (49.6%)	140 (41.5%)	

Association of participants’ profile with maternal undernutrition

Out of all the women who were found to be underweight, 84.7% belonged to the 18-25 years age group, although there was no significant association between age group and presence of undernutrition (p-value=0.125). Most of the underweight women followed the Hindu religion (41.7%), and came from a non-tribal background (55.6%), and were housewives (81.9%) belonging to a lower-middle-class family. However, these results were not significant statistically. From among the socio-demographic variables, only family type was found to have a significant association (p-value=0.020) with maternal undernutrition, with 93.1% of undernourished pregnant women belonging to joint families (Table 3).

**Table 3:** Association of study variables with maternal undernutrition (n=284) Test of significance: Pearson's chi-square test BMI: Body mass index, BG: Brahm Govind, MDD-W: Minimum dietary diversity-Women * Statistically significant

Socio-demographic variables		Nutritional status based on BMI			
		Undernutrition (n=72)	Normal (n=212)	Total (n=284)	p-value
Age group	18-25 years	61 (84.7%)	153 (72.2%)	214 (75.4%)	0.125
	26-30 years	11 (15.3%)	51 (24.1%)	62 (21.8%)	
	31-35 years	0	7 (3.3%)	7 (2.5%)	
	36-40 years	0	1 (0.5%)	1 (0.4%)	
Religion	Hindu	30 (41.7%)	90 (42.5%)	120 (42.3%)	0.609
	Sarna	27 (37.5%)	66 (31.1%)	93 (32.7%)	
	Muslim	15 (20.8%)	54 (25.5%)	69 (24.3%)	
	Christian	0	2 (0.9%)	2 (0.7%)	
Ethnicity	Non-tribal	40 (55.6%)	127 (59.9 %)	167 (58.8%)	0.517
	Tribal	32 (44.4.%)	85 (40.1%)	117 (41.2%)	
Educational status	Post-graduate	0	5 (2.4%)	5 (1.8%)	0.172
	Graduate	7 (9.7%)	17 (8.0%)	24 (8.5%)	
	Intermediate	14 (19.4%)	62 (29.2%)	76 (26.8%)	
	Matriculate	24 (33.3%)	69 (32.5%)	93 (32.7%)	
	Up to high school	23 (31.9%)	51 (24.1%)	74 (26.1%)	
	Literate but no formal education	4 (5.6%)	4 (1.9%)	8 (2.8%)	
	Illiterate	0	4 (1.9%)	4 (1.4%)	
Occupation	Housewife	59 (81.9%)	188 (88.7%)	247 (87.0%)	0.234
	Student	4 (5.6%)	5 (2.4%)	9 (3.2%)	
	Employed with private firm	5 (6.9%)	12 (5.7%)	17 (6.0%)	
	Teacher	2 (2.8%)	4 (1.9%)	6 (2.1%)	
	Farmer	0	1 (0.5%)	1 (0.4%)	
	Labor	0	2 (0.9%)	2 (0.7%)	
	PRI member	1 (1.4%)	0	1 (0.4%)	
	Anganwadi worker	1 (1.4%)	0	1 (0.4%)	
Family type	Joint	67 (93.1%)	173 (81.6%)	240 (84.5%)	0.020*
	Nuclear	5 (6.9%)	39 (18.4%)	44 (15.5%)	
Socio-economic status based on updated BG Prasad Classification Scale (2024)	Class I (Upper)	2 (2.8%)	4 (1.9%)	6 (2.1%)	0.761
	Class II (Upper middle)	7 (9.7%)	22 (10.4%)	29 (10.2%)	
	Class III (Middle)	21 (29.2%)	79 (37.3%)	100 (35.2%)	
	Class IV (Lower middle)	34 (47.2%)	87 (41.0%)	121 (42.6%)	
	Class V (Lower)	8 (11.1%)	20 (9.4%)	28 (9.9%)	
Dietary variables					
Dietary pattern	Vegetarian	23 (31.9%)	43 (20.3%)	66 (23.2%)	0.043*
	Non-vegetarian	49 (68.1%)	169 (79.7%)	218 (76.8%)	
Dietary diversity score based on MDD-W	Adequate	26 (36.1%)	134 (63.2%)	160 (56.3%)	<0.001*
	Inadequate	46 (63.9%)	78 (36.8%)	124 (43.7%)	

Association of participants’ profile with maternal overnutrition

The proportion of women screened with overnutrition was found to be equal in the 18-25 and 26-30-years age (45.3% each) groups, with a minor proportion in later years (9.4%) (p-value=0.002). Most of the overnourished women were followers of the Muslim religion (45.3%), followed by Hindus (39.6%) (p-value=0.017). Both of these results were statistically significant. Most of the women who were detected to have overnutrition came from a non-tribal background (73.6%), were educated up to intermediate level (34.0%), worked as housewives (98.1%), and resided in joint families (86.8%) belonging to middle-class households (39.6%). However, these results were statistically non-significant (Table 4).

**Table 4:** Association of study variables with maternal overnutrition (n=265) Test of significance: Pearson's chi-square test BMI: Body mass index, BG: Brahm Govind, MDD-W: Minimum dietary diversity-Women * Statistically significant

Socio-demographic variables		Nutritional status based on BMI			
		Normal (n=212)	Overnutrition (n=53)	Total (n=265)	p-value
Age group	18-25 years	153 (72.2%)	24 (45.3%)	177 (66.8%)	0.002*
	26-30 years	51 (24.1%)	24 (45.3%)	75 (28.3%)	
	31-35 years	7 (3.3%)	5 (9.4%)	12 (4.5%)	
	36-40 years	1 (0.5%)	0	1 (0.4%)	
Religion	Hindu	90 (42.5%)	21 (39.6%)	111 (41.9%)	0.017*
	Sarna	66 (31.1%)	8 (15.1%)	74 (27.9%)	
	Muslim	54 (25.5%)	24 (45.3%)	78 (29.4%)	
	Christian	2 (0.9%)	0	2 (0.8%)	
Ethnicity	Non-tribal	127 (59.9%)	39 (73.6%)	166 (62.6%)	0.066
	Tribal	85 (40.1%)	14 (26.4%)	99 (37.4%)	
Educational status	Post-graduate	5 (2.4%)	3 (5.7%)	8 (3.0%)	0.528
	Graduate	17 (8.0%)	6 (11.3%)	23 (8.7%)	
	Intermediate	62 (29.2%)	18 (34.0%)	80 (30.2%)	
	Matriculate	69 (32.5%)	13 (24.5%)	82 (30.9%)	
	Up to high school	51 (24.1%)	11 (20.8%)	62 (23.4%)	
	Literate but no formal education	4 (1.9%)	0	4 (1.5%)	
	Illiterate	4 (1.9%)	2 (3.8%)	6 (2.3%)	
Occupation	Housewife	188 (88.7%)	52 (98.1%)	240 (90.6%)	NaN
	Student	5 (2.4%)	1 (1.9%)	6 (2.3%)	
	Employed with private firm	12 (5.7%)	0	12 (4.5%)	
	Teacher	4 (1.9%)	0	4 (1.5%)	
	Farmer	1 (0.5%)	0	1 (0.4%)	
	Labor	2 (0.9%)	0	2 (0.8%)	
	PRI member	0	0	0	
	Anganwadi worker	0	0	0	
Family type	Joint	173 (81.6%)	46 (86.8%)	219 (82.6%)	0.372
	Nuclear	39 (18.4%)	7 (13.2%)	46 (17.4%)	
Socio-economic status based on updated BG Prasad Classification Scale (2024)	Class I (Upper)	4 (1.9%)	2 (3.8%)	6 (2.3%)	0.657
	Class II (Upper middle)	22 (10.4%)	8 (15.1%)	30 (11.3%)	
	Class III (Middle)	79 (37.3%)	21 (39.6%)	100 (37.7%)	
	Class IV (Lower middle)	87 (41.0%)	17 (32.1%)	104 (39.2%)	
	Class V (Lower)	20 (9.4%)	5 (9.4%)	25 (9.4%)	
Dietary variables					
Dietary pattern	Vegetarian	43 (20.3%)	3 (5.7%)	46 (17.4%)	0.012*
	Non-vegetarian	169 (79.7%)	50 (94.3%)	219 (82.6%)	
Dietary diversity score based on MDD-W	Adequate	134 (63.2%)	37 (69.8%)	171 (64.5%)	0.369
	Inadequate	78 (36.8%)	16 (30.2%)	94 (35.5%)	

Binomial logistic regression for predictors of maternal malnutrition, undernutrition, and overnutrition

On applying binomial logistic regression to socio-demographic variables that were found to be statistically significant in the chi-square tests of association, it was found that family type and inadequacy of dietary diversity were significant risk factors for both maternal malnutrition and undernutrition, while religion, particularly being from a Muslim community was a significant risk factor for maternal overnutrition. With regards to dietary patterns, following a vegetarian diet was a risk factor for maternal undernutrition, while following a non-vegetarian diet was a risk factor for maternal overnutrition (Table 5).

**Table 5:** Binomial logistic regression for predictors of maternal malnutrition, undernutrition and overnutrition CI: Confidence interval, MDD-W: Minimum dietary diversity-Women * Statistically significant

Variable		Odds ratio	95% CI for odds ratio		p-value
			Lower	Upper	
Binomial logistic regression for predictors of maternal malnutrition (n=337)					
Family type	Joint	2.123	1.066	4.228	0.032*
	Nuclear	1			
Dietary diversity score based on MDD-W	Inadequate	1.691	1.080	2.647	0.022*
	Adequate	1			
Binomial logistic regression for predictors of maternal undernutrition (n=284)					
Family type	Joint	3.021	1.142	7.991	0.026*
	Nuclear	1			
Dietary pattern	Vegetarian	1.845	1.015	3.354	0.045*
	Non-vegetarian	1			
Dietary diversity score based on MDD-W	Inadequate	3.039	1.743	5.300	<0.001*
	Adequate	1			
Binomial logistic regression for predictors of maternal overnutrition (n=265)					
Religion	Muslim	2.421	1.299	4.514	0.005*
	Other	1			
Dietary pattern	Non-vegetarian	4.240	1.216	14.252	0.019*
	Vegetarian	1			

## Discussion

The observations and findings from the study revealed that overall, 21.4% of women were underweight, 14.8% were overweight, 0.9% were obese, and 62.9% of women were in the normal weight range based on Body Mass Index (BMI). Family type, dietary habits, and community practices were found to be significant determinants of nutritional status among pregnant women.

An urban hospital-based study by Sen et al. in 2010 in Siliguri, West Bengal, India, among 503 pregnant women found the proportions of underweight, normal, and overweight to be 9.94%, 75.75%, and 14.31% [14]. The proportion of underweight in Sen et al. is found to be lower than in this study, which can be explained by the urban nature of the study where overnutrition rather than undernutrition is more prevalent.

A study done by Madhavi and Singh in rural Karnataka in 117 pregnant women in 2011 found the percentage of underweight, normal, and overweight women to be 23.9%, 71.8%, and 4.3%, respectively, which is similar to our study, though also reflects the increasing burden of overnutrition in rural India [15]. Jharkhand, being a comparatively poorer state than Karnataka and also considering the time gap between the studies, still has a higher proportion of underweight and a lower proportion of overweight as per NFHS-5 data. While the proportion of underweight women in the 15-49 age group stands at 17.2% for Karnataka, it is higher in Jharkhand at 26.2%. On the other end of the spectrum, the percentage of overweight women in Karnataka is higher at 30.2%, compared to Jharkhand’s 11.9%. While on the one hand, this reflects the slow rate of socioeconomic progress in Jharkhand, on the other hand, this also reflects the increasing burden of overnutrition in Karnataka.

A study done by Joshi et al. in 2017 among 200 pregnant women in rural settings in the outskirts of Bhopal found the proportion of underweight, normal, and overweight women to be 25%, 68%, and 7%, respectively, which is comparable to our study in some regards [11]. While the proportion of underweight was higher, that of overweight was lower compared to our study. Since the said study was conducted in 2017, an analysis of NFHS-4 (2016-17) and NFHS-5 (2019-21) needs to be done to understand these findings. As per NFHS-4, Madhya Pradesh had a higher proportion of underweight women of reproductive age (28.4%) compared to the recent prevalence (26.2%) of underweight among the same in Jharkhand according to NFHS-5. The finding of the lower percentage of overweight in the said study compared to our study is paradoxical as the proportion of overweight as per NFHS-4 in Madhya Pradesh was higher (13.6%) compared to that in Jharkhand as per recent estimates (11.9%). This finding could be explained through the time gap of almost six years between the two studies, suggesting the chances of an epidemiological and nutritional transition having occurred during the time.

The percentage of underweight and normal pregnant women in this study was higher compared to a study by Bhavadharini et al. in Chennai in 2017, where it was 5.6% and 29%, respectively, out of 2728 pregnant women [16]. The proportion of overweight and obese was, however, higher in that study at 18.5% and 46.9%, respectively. This difference can be explained by the pre-existing difference in BMI of women in the reproductive age (15-49 years) between the states of Tamil Nadu and Jharkhand based on NFHS-5 data. While Jharkhand has a mean BMI of 21.0 kg/m2, Tamil Nadu has a higher mean BMI of 24.3 kg/m^2^. The proportion of underweight women in Jharkhand is 26.2%, which is higher compared to Tamil Nadu’s 12.6%. The situation is reversed for the proportion of overweight women, which in Jharkhand is lower at 11.9% compared to 40.5% in Tamil Nadu.

Gokhale et al., in a study done on 204 pregnant women in rural areas of Pune in 2021, found 33.8% of women to be underweight [17]. This is higher than the 21.4% found in this study. This finding needs further research for a proper explanation.

A study done amongst 509 pregnant urban slum dwellers in Pune by Deshpande et al. in 2022 using Asian cut-offs of BMI found 28% of women to be underweight and 25% of women to be overweight [18]. This is higher than that found in our study in both regards. With regards to underweight, this finding is greater than the finding in NFHS-5, which reported 13.3% underweight in women of reproductive age in urban areas, lower than the 21.3% reported for rural areas. As for overnutrition, the NFHS-5 reported 33.3% overweight women in the same group in urban areas, which is expected when compared to our study, which was done in a mixed residential group. Urban slum dwellers mostly belong to lower socio-economic groups and tend to have lower dietary diversity. On the one hand, they are at risk of undernutrition due to their lower socio-economic status, while on the other hand, they also face the risk of overnutrition due to the abundance of packed food items and fast food, which provide nutrient-poor calories. Hence, further studies need to be undertaken to understand the differences in nutritional status of urban slum and non-slum residents.

An adequate dietary diversity score was found to be a protective factor of maternal undernutrition. Dietary diversity score is a proxy indicator of nutrient adequacy [19] so having a diverse diet ensures that the nutrient requirements of the body are met, thus resulting in a good nutritional status. A similar finding was reported in Gelebo et al. [20], Desyibelew et al. [21], Saraswat et al. [22], and Diddana et al. [23] studies.

In this study, living in a joint family was found to be a risk factor for maternal undernutrition. This could be due to the division of resources, specifically food, among more members of the family, particularly male members who are considered to be the breadwinners of the joint families in traditional Indian settings. The decision-making power regarding food habits is also not bestowed upon female members in such settings. A similar finding was reported by Gelebo et al. [20] and Kedir et al. [24].

Our study found a non-vegetarian diet to be a risk factor for overnutrition. This is similar to the finding reported by Rizzo et al., which reported that non-vegetarians had higher BMI values than vegetarians [25]. The explanation for this is that energy-dense nutrients such as total fat, saturated fat, and trans-fat are likely to be higher in a non-vegetarian diet. Our study also found women belonging to the Muslim community to be overweight. This could be due to the higher prevalence of non-vegetarianism found in the Muslim culture.

Strengths of the study

A good sample size was taken by simple random sampling for the conduct of this study. A pre-tested semi-structured questionnaire was administered to the study participants to collect data about the different variables, which ensured the accuracy of the data. Good quality equipment was used to collect the anthropometric data after proper calibration before every session. By focusing on the issue of malnutrition among pregnant women in a specific region of Jharkhand, the study has tried to address a highly relevant issue for public health interventions in the area.

Limitations of the study

While the study focuses on prevalence, it may not capture the full spectrum of factors influencing maternal malnutrition, such as socio-economic factors, quantitative assessment of dietary habits, and healthcare access. The study used the BMI scale to measure maternal malnutrition, which is not a widespread tool to assess malnutrition among pregnant women. Even the few authorities who recommend its use, mention its use before 20 weeks’ gestation [26,27]. Ideally, weight gain during each trimester on the basis of pre-pregnancy BMI should have been taken to measure malnutrition based on the recommendation of the Institute of Medicine (IOM), USA [28], but it would have been difficult to trace and follow-up would-be mothers from the community. Recall methods were used for dietary assessment, which carry the risk of recall bias.

## Conclusions

The present study found more than a third of pregnant women suffering from malnutrition, reflecting the double burden of malnutrition in our study setting. Factors such as socio-demographic dynamics and dietary habits were found to be significant contributors in this regard. With the epidemiological and nutritional transition occurring in India, it is important that appropriate interventions be initiated at the community, family, and individual levels to address this issue. Generating awareness, providing health education, tackling socio-economic issues, promoting family support, and tailoring healthcare facilities to focus on this problem can prove to be crucial steps to contend with the challenges posed by malnutrition.
